# Reducing care burden and improving adherence to health-promoting behaviors among family caregivers of patients with multiple sclerosis through a healthy lifestyle empowerment program

**DOI:** 10.1186/s12912-022-00961-8

**Published:** 2022-08-16

**Authors:** Abdolsamad Homayouni, Parvaneh Vasli, Fatemeh Estebsari, Maliheh Nasiri

**Affiliations:** 1 Department of Community Health Nursing, School of Nursing and Midwifery, Shahid Beheshti University of Medical Sciences, Tehran, Iran; 2 Department of Basic Sciences, School of Nursing and Midwifery, Shahid Beheshti University of Medical Sciences, Tehran, Iran

**Keywords:** Multiple sclerosis, Care burden, Health behaviors, Empowerment, Caregiver

## Abstract

**Background and aim:**

The chronic, progressive nature of multiple sclerosis (MS) demands long-term family-centered care for patients. In view of that, inadequate education and support provided for the family caregivers (FCGs) of MS patients increase their care burden (CB) and affect their lifestyle. This study aimed to investigate the impact of a healthy lifestyle empowerment program (HLEP) on CB and adherence to health-promoting behaviors (HPBs) in the FCGs of patients suffering from MS.

**Methods:**

In this experimental study with parallel groups, conducted in Iran in 2020, a total of 60 FCGs of MS patients were recruited, and then randomized into intervention (*n* = 30) or control (*n* = 30) groups. The intervention program, the HLEP, was thus implemented virtually via WhatsApp in the intervention group upon coordinating with the MS Association in the city of Yasuj, Iran, and selecting the participants. The data were collected at three stages, including baseline, follow-up 1 (immediately after the HLEP), and follow-up 2 (three months after HLEP). The research tools were a 14-item demographic survey questionnaire, the 24-item Caregiver Burden Inventory, and the 52-item Health-Promoting Lifestyle Profile-II. Independent-samples t-test, repeated measures analysis of variance, and a linear mixed model were further used for statistical analyses, considering the significant level of 0.05.

**Results:**

The study results revealed a significant decrease in the CB scores from the baseline to the follow-up 2 (77.03 ± 15.76 to 42.33 ± 12.37), and a significant increase in the values of adherence to HPBs from the baseline to the follow-up 2 (123.53 ± 14.01 to 148.06 ± 15.04) were obtained in the intervention group (*p* < 0.001). The linear mixed model also showed that the significant absolute changes in the scores of CB and adherence to HPBs during the follow-ups in the intervention group, compared to those in the controls, were − 8.92 and 16.47 units, respectively (*p* < 0.001).

**Conclusion:**

Health care managers, planners, and providers are highly recommended to start developing and implementing various HLEPs for reducing CB and improving adherence to HPBs among the FCGs of patients with MS.

## Introduction

Multiple sclerosis (MS) is known as one of the autoimmune chronic diseases, with a demyelinating inflammatory pathology. This health condition affects the central nervous system, including the cerebral peduncles as well as the periventricular areas of the brain, the optic nerves, and the spinal cord [1, 2]. MS often occurs in young adults, namely those aged 20–40 [3]. The mean age of MS patients is 30, and this disease appears in women by a ratio of 2 (or 3) to 1 over men [4, 5]. The related statistics show that 2.8 million people are living with MS across the world, with the prevalence rate of approximately 0.6% in Iran [5, 6].

MS also includes various symptoms, such as visual problems, muscle weakness, extreme fatigue, cognitive impairment, overheating, speech disorders, depression, and frustration [4, 5]. Considering its disability problems, about a quarter of patients with MS require vast long-term care, and 30% of them must receive home care. Informal caregivers, such as spouses, family members, or other relatives, provide 80% of home care for such patients [3, 7].

Chronic conditions such as MS can affect the lifestyle of patients and their families simultaneously [7]. Family caregivers (FCGs) have to adapt to the presence of MS and other unpredictable events, such as physical and mental problems, over time [1]. Of note, FCGs broadly represent the individuals who take on unpaid caring roles, and provide emotional, physical, or practical support in response to an illness, disability, or age-related needs [8].

Previous studies have indicated a significant positive association between the degree of MS-related disability in patients and care burden (CB) [2]. A large body of literature accordingly has suggested that the caregivers of patients with MS, and those caring for cases affected with other chronic illnesses, are at risk of undergoing considerable CB [9]. Here, CB can be defined as the strain experienced by a person who cares for a chronically ill, disabled, or elderly family member [10].

CB also contributes to lifestyle changes, which result in depression, anxiety, low physical health, and social isolation among FCGs [11]. It has been further associated with poor self-care practices, and increased risk for physical illnesses in FCGs [12]. In other words, the health-promoting behaviors (HPBs) of FCGs can be negatively affected by caring roles and CB [13], especially in Asian countries, wherein FCGs often assume the full responsibility of caring for other family members [14]. In this respect, HPBs involve activities and habits that lead to the improvement of various dimensions of people’s health [15]. Such behaviors include six dimensions, viz. health responsibility, physical activity, nutrition, spiritual growth, interpersonal relationships, and stress management [16]. FCGs who are engaged in HPBs can thus prevent or minimize the likelihood of developing illnesses via direct and indirect effects of such behaviors [12].

More attention should be accordingly paid to CB with regard to the changes in lifestyle and the improvements in HPBs [17]. The full awareness of the CB features and its association with HPBs among the FCGs of patients living with MS can thus demand particular intervention programs to improve healthy lifestyles [3].

Although it is vital to support and empower FCGs, these caregivers have inadequate interactions with health care team members, and are not even provided with the information they need for their safety, lifestyle, and well-being [18]. The importance of lifestyle largely stems from its influence on quality of life and disease prevention [19]. Lifestyle refers to the day-to-day activities that individuals accept as part of their life, affecting their health status [20]. A healthy lifestyle accordingly means changing unhealthy habits and developing healthy ones, while engaging in healthy activities and behaviors [19].

Based on various studies with different populations, it seems that one of the best methods to reduce CB and boost adherence to health-promoting behaviors (HPBs) in FCGs is education and rehabilitation programs [19, 21, 22]. The documented positive effect of educational programs for FCGs on patients’ functional improvement and their satisfaction as well as the possible influence of such primary caregivers on patients’ health outcomes are thus noticeable [23]. Two studies in Iran had accordingly investigated the effect of group-based psychological training programs and mindfulness-based intervention via the Internet on the CB of the FCGs of patients with MS [24, 25]. Martindale-Adams et al. had also reflected on the effect of a validated, behavioral, caregiving intervention program on CB, depression, anxiety, and challenging MS behaviors in the FCGs of cases living with MS. Besides, Lök and Bademli had examined the effects of the “First You Should Get Stronger” program on CB and HPBs in the FCGs of dementia patients. As well, Farran et al. had studied the effect of a physical activity intervention program on physical activity and perceived CB among the FCGs of patients with dementia [26].

Although several studies have been so far conducted on the effect of different interventions on CB and adherence to some dimensions of HPBs in the FCGs of patients with MS and dementia in Iran and other countries, none has specifically focused on assessing the impact of the healthy lifestyle promoting program (HLEP) on variables such as CB and adherence to HPBs among the FCGs of MS patients in Iran. Empowerment is here defined as positively controlling a person’s mind and body, developing a positive attitude, and actively trying to understand one’s role as a caregiver to promote a family’s caregiving abilities. The other features of empowerment include centering on others as well as oneself, providing assistance to care receivers to promote their quality of life, and creating constructive relationships with others [27]. Accordingly, this study aimed to investigate the effect of the HLEP on CB and adherence to HPBs in the FCGs of MS patients. For this purpose, two hypotheses were raised as follows:

- *H*_*1*_: The HLEP affects CB in the FCGs of patients with MS.
- *H*_*2*_: The HLEP affects adherence to HPBs in the FCGs of patients with MS.

## Methods

### Study design and setting

This experimental, single-blinded study, with parallel groups, was fulfilled in 2020 at the MS Association in the city of Yasuj, Iran. The participants were blinded to the group allocation and the intervention program so as not to affect their behaviors.

### Participants and recruitment

The study participants were the FCGs of patients with MS. The sample size was thus determined as follows:

$$\mathrm n=\frac{2\hspace{0.25em}\left({\mathrm Z}_\frac\alpha2+{\mathrm Z}_{1-\beta}\right)^2\hspace{0.25em}\mathrm\sigma^2}{\left({\mathrm\mu}_1-{\mathrm\mu}_2\right)^2},\hspace{0.25em}\mathrm\alpha=0.05,\hspace{0.25em}\mathrm\beta=0.10,\frac{{\mathrm\mu}_1-{\mathrm\mu}_2}{\mathrm\sigma}=0.75$$

The mean (μ) and variance (σ) values were accordingly obtained based on the study conducted by [24]. Upon assuming the 10% dropout rate, the required sample size in each group was equal to 30.

$$\mathrm{n}=2{\left(1.96+0.85\right]}^2{\left(\frac{1}{0.75}\right]}^2=27,\mathrm{dropout}\ \mathrm{rate}=10\%,\kern0.5em \mathrm{n}=30$$

The list of the patients as the members of the MS Association in the city of Yasuj was provided to the researcher. The researcher then used the membership list to select the FCGs of MS patients, using coin tosses, and randomized them into either the intervention or control groups. Afterward, each of the selected FCGs was contacted, and the inclusion criteria were reviewed. The sampling process continued until the required number of the FCGs in each group was obtained. Of note, the inclusion criteria were the age range of 18 to 60, being the primary FCGs of patients with MS (i.e., the one as the most responsible for the care of the patients with MS, such as father, mother, sibling, spouse, or child), patient care for at least six months, no diagnosis of psychiatric disorders, no restrictions on nutrition and physical activity due to certain diseases, such as diabetes or arthritis, no simultaneous participation in similar training programs, and using smartphones and apps, like WhatsApp; on the other hand, the exclusion criteria were showing reluctance to participate in the study and being absent for more than one educational session.

**Fig. 1:**
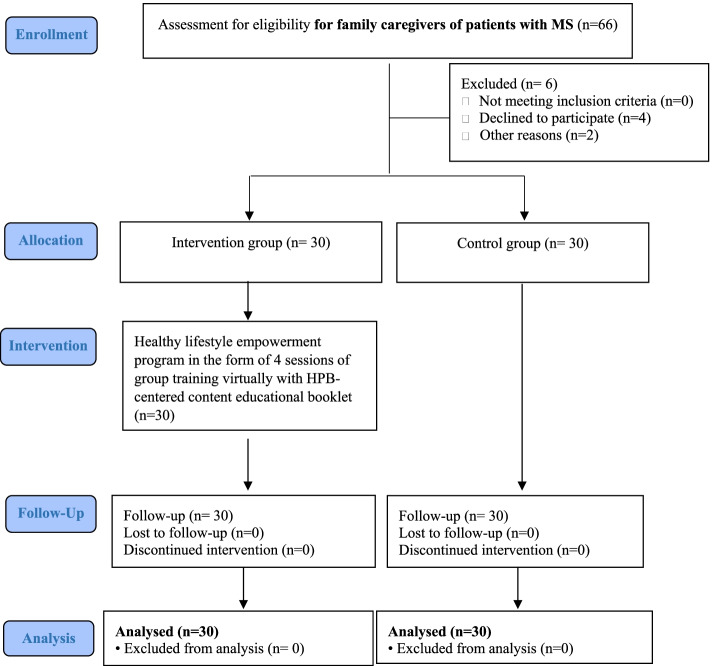
CONSORT flow chart of the study

Initially, 33 eligible FCGs of MS patients were included in the intervention and control groups equally (totally 66 FCGs). However, three FCGs in the intervention group did not desire to continue participating in the study, two FCGs in the control group failed to complete the research tools, and one of them withdrew from the study. The CONSORT flow chart is illustrated in Fig. 1.

### HLEP

Based on its schedule, the HLEP was implemented by the first researcher in four educational sessions (lasting 45–60 minutes) for the intervention group. The FCGs in the control group, however, did not receive the program during the study. The educational program content was also provided based on the recent studies and with the collaboration of experts working in the fields of nutrition, religion, and psychology, using the dimensions of HPBs, including health responsibility, physical activity, nutrition, spiritual growth, interpersonal relationships, and stress management [28–31]. The expert opinions were also obtained from four faculty members from Department of Community Health Nursing (*n* = 2), Department of Psychiatric Nursing (*n* = 1), and Department of Medical-Surgical Nursing (*n* = 1), who were specialized in working with the families of patients with MS. The final HLEP content was presented in accordance with the expert opinions. Table 1 shows the schedule.

**Table 1:** The HLEP for the FCGs of Patients with MS in the Intervention Group

Sessions	Objectives	Content
One	**Improving HPBs in the health responsibility dimension**	***Health responsibility*: MS disease, treatment, care for patients with MS, role of caregivers in improving and maintaining individuals’ health, CB possibility, importance of self-care, enhancing self-efficacy in patients with MS during self-care, role of caregivers to choose a healthy lifestyle**
Two	**Improving HPBs in the physical activity and nutrition dimensions**	***Physical activity*: Benefits of increasing physical activity quality and quantity, simple ways to increase physical activity, exercise programs** ***Nutrition*: An introduction to food groups, an introduction to MyPlate, principles of healthy cooking, avoiding unsaturated fatty acids, avoiding weight gain**
Three	**Improving HPBs in the spiritual growth, interpersonal relationships, and stress management dimensions**	***Spiritual growth*: Impact of prayer on physical and mental health, role of spirituality in facilitating treatment, impact of worshipping rituals and spiritual beliefs, ways to achieve peace of mind** ***Interpersonal relationships*: Communication methods, factors affecting personal relationships, benefits of interpersonal relationships for caregivers** ***Stress management*: Definition of stress, causes of stress, teaching simple techniques to deal with stress**
Four	**Improving all dimensions of HPBs**	**Reviewing previous content, Q&A, discussion, presenting an educational booklet, videos, and clips**

*HLEP* Healthy lifestyle empowerment program, *FCGs* Family caregivers, *MS* Multiple Sclerosis, *CB* Care burden, *HPBs* Health-promoting behaviors

Due to the coincidental occurrence of the data collection and the implementation of the intervention program with the outbreak of the coronavirus disease 2019 (COVID-19) pandemic, all the research steps were performed virtually. Accordingly, after coordinating with the MS Association in the city of Yasuj and selecting the participants, a separate group was created in WhatsApp for both groups (viz. intervention and control), and all three stages of data collection and HLEP implementation (for the intervention group) were guided through this messenger.

Ethical considerations, i.e., the introduction of the researcher and the study objectives, the voluntary basis of participation in the study, the possibility of leaving the study at any stage, and the confidentiality of information were also reminded to the participants. For the intervention group, the educational content was uploaded in the form of a booklet as well as videos and clips. To strengthen the intervention effect, reminders about the educational content were presented four times in the intervention group after the program was over, viz. two, four, six, and eight weeks later. Moreover, the education and care processes were evaluated while answering the participants’ questions.

### Baseline and follow-ups

The changes in the intervention outcomes were defined as the differences from the baseline to the follow-up 1 (immediately after the HLEP implementation) and the follow-up 2 (three months after its completion) stages of CB and adherence to HPBs. Considering the COVID-19 pandemic and the participants’ health maintenance, the online research tools were provided by the first author. The questionnaire link was then sent to the WhatsApp accounts of both intervention and control groups, and they were asked to complete the tools the day before the intervention.

The follow-up measurements were also completed immediately and three months after implementing the HLEP, and the FCGs were asked to complete the research tools online.

### Measures

In this study, the data were collected, using three tools, including a demographic survey questionnaire, the Caregiver Burden Inventory (CBI), and the Health-Promoting Lifestyle Profile-II (HPLP-II).

#### Demographic survey questionnaire

The demographic survey questionnaire for FCGs of patients with MS contained 6 items, including age, gender, marital status, education, occupation, and **relationship** with the patient.

#### CBI

CB was assessed using the CBI, provided by Novak and Guest in 1989 [17]. The 24-item CBI as a multi-dimensional tool was comprised of five subscales, i.e., time dependence (items 1–5), developmental burden (items 6–10), physical burden (items11–14), emotional burden (items 15–19), and social burden (items 20–24). All items were scored using a five-point Likert-type scale, ranged from never = 0 to almost always = 5 [5]. Accordingly, the CBI total score ranged from 24 to 120. Its Persian version was thus used in this study. Of note, the Cronbach’s alpha coefficient of the CBI for Iranian patients with stroke was 0.91 [22]. In the present study, this value was 0.929.

#### HPLP-II

Adherence to HPBs was measured via the HPLP-II, developed by Walker et al. in 1987 [16]. It contained 52 items and six subscales, viz. health responsibility (nine items), spiritual growth (nine items), physical activity (eight items), interpersonal relationships (nine items), nutrition (nine items), and stress management (eight items). The items were also scored based on a four-point Likert-type scale as 1 = never, 2 = sometimes, 3 = often, and 4 = routinely. The total score of the HPLP-II was computed by the mean value of all 52-items, and ranged from 52 to 208. The higher value of the total score accordingly represented better health behaviors. In the original version of the HPLP-II, the Cronbach’s alpha coefficient of the overall scale was 0.94, and that was 0.79–0.87 for six subscales [16]. This value for the Persian version of the HPLP-II was 0.89 for women with heart failure [32]. In this study, the Cronbach’s alpha coefficient of 0.896 was obtained for the HPLP-II.

### Statistical analysis

The study data were examined for normality distribution and missing values. Descriptive statistics, that is mean ± standard deviation (SD) and frequency/percentage, were also employed for the continuous and categorical variables, respectively. Chi-square test, Fisher’s exact test, and Mann-Whitney U test were further exploited to compare the demographic characteristics in both groups. To compare the within-group changes in the CBI and HPLP-II values from the baseline to the follow-up 1 & 2 stages, repeated measures analysis of variance (ANOVA) was utilized. Independent-samples t-test was then performed to compare the between-group changes in the CBI and HPLP-II scores. Finally, a linear mixed model was applied to determine the absolute changes in the CBI and HPLP-II from the baseline to the follow-up stage, by controlling the demographic characteristics as a confounding factor. The statistical analysis was conducted using the SPSS software package (ver. 22). As well, *p* < 0.05 was considered for the statistical significance level.

## Results

### Participants’ baseline information

A total of 60 FCGs of patients living with MS (30 cases in each group) participated in this study, with the response rate of 100%. The age mean ± SD of the FCGs of patients with MS in the intervention and control groups were 32.23 ± 6.53 and 36.90 ± 9.34, respectively. Other demographic characteristics of the participants are presented in Table 2. There was also no statistical difference between both groups in terms of the demographic characteristics of the FCGs.

**Table 2:** Demographic information of the patients with MS and their FCGs

Group	Intervention group (***n*** = 30)		Control group (***n*** = 30)		***p***-value
Variables	Frequency	Percentage	Frequency	Percentage	
**Gender**					
Female	14	46.7	11	36.7	0.60^b^
Male	16	53.3	19	63.3	
**Marital status**					
Single	4	13.3	4	13.3	0.60^a^
Married	26	86.7	25	83.4	
Widow/ Divorced	0	0	1	3.3	
**Education**					
Illiterate/ Primary school	1	3.4	6	20	0.13^a^
Middle school	2	6.6	2	6.6	
High school/ diploma	12	40	6	20	
University degree	15	50	16	53.3	
**Occupation**					
Homemaker	10	33.3	8	26.7	0.12^a^
Unemployed	7	23.3	9	30	
Self-employed	1	3.3	1	3.3	
Employee	12	40	12	40	
**Relationship with the patient**					
Mother	2	6.7	2	6.7	0.12^a^
Sister	4	13.3	1	3.33	
Brother	4	13.3	1	3.33	
Child	2	0.3	5	16.7	
Spouse	18	60	21	70	

*FCGs* Family caregivers, *MS* Multiple Sclerosis

^a^Chi-square test

^b^Fisher’s exact test’

### Changes in CB in intervention and control groups

**Fig. 2:**
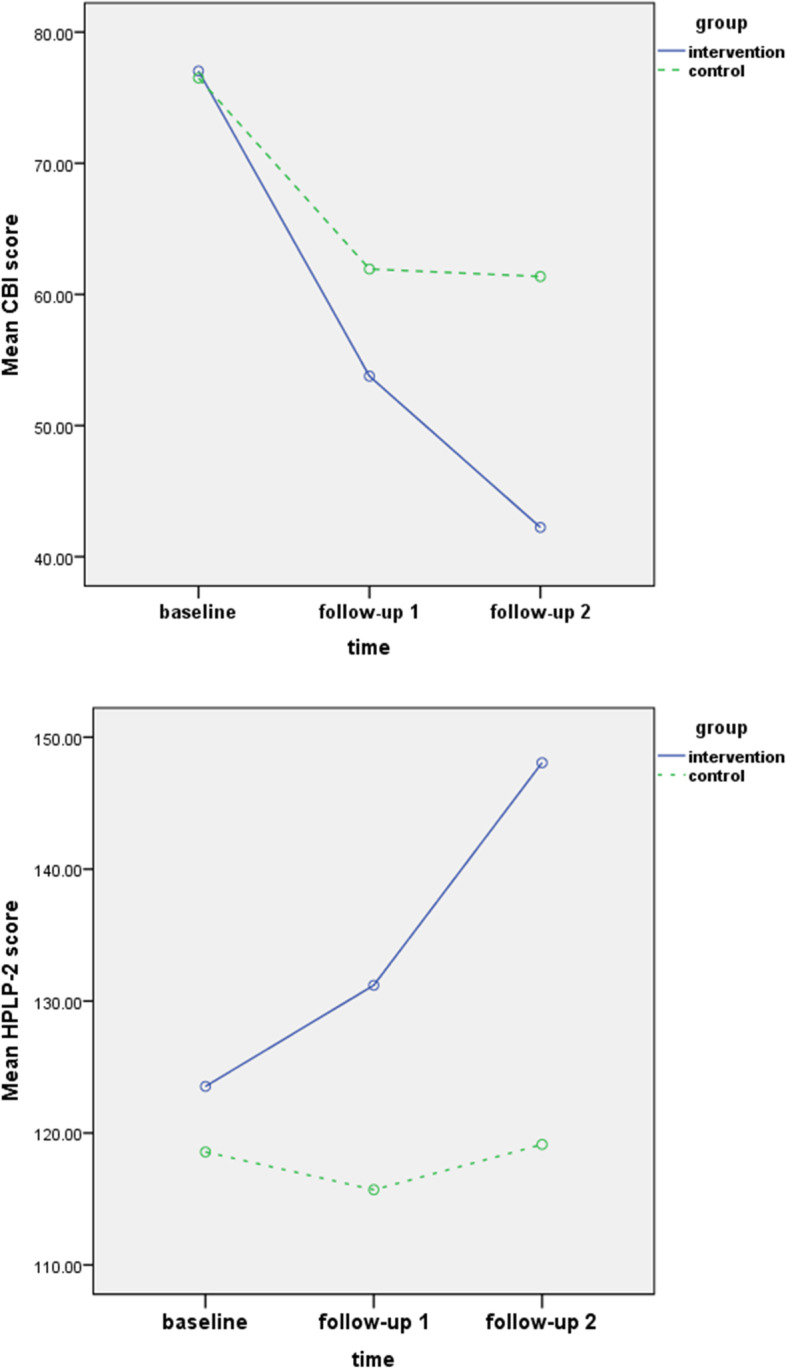
Changes in CBI and HPLP-2

The results of within-group and between-group changes in the CBI scores from the baseline to the follow-up 1 & 2 stages are given in Table 3 and Fig. 2. Independent-samples t-test results accordingly showed no significant difference between the CBI scores (viz. total and individual dimensions) of both groups at the baseline stage (*p* > 0.05). Repeated measures ANOVA outcomes further indicated that the mean values of the CBI total score and other dimensions in the intervention group were significantly lower during the follow-up 1 & 2 stages (*p* < 0.001). The within-group changes in the intervention group in all scores were also significant (*p* < 0.001), except for the emotional burden (*p* = 0.13). The within-group changes in the CBI scores in the control group were correspondingly significant (*p* < 0.001). Besides, the within-group comparison demonstrated a significant decrease in the CBI scores in the intervention group, so that the total mean score dropped from 77.03 ± 15.76 to 42.33 ± 12.37.

**Table 3:** Comparison of changes in mean CB of the intervention and control groups in FCGs of patients with MS

Group		Intervention (*n* = 30)	Control (*n* = 30)	Intervention vs. Control ^a^
CBI (total and individual dimensions)				
Total CBI	Baseline	77.03 ± 15.76	76.50 ± 14.78	0.89
	Follow-up 1	53.77 ± 5.35	61.93 ± 16.09	< 0.001
	Follow-up 2	42.33 ± 12.37	61.37 ± 7.15	< 0.001
	Follow-ups vs. Baseline^b^	0.001	0.001	
Time-dependence burden	Baseline	16.80 ± 3.87	16.40 ± 3.06	0.63
	Follow-up 1	12 ± 2.01	13.03 ± 3.95	< 0.001
	Follow-up 2	9.46 ± 4.01	11.37 ± 2.47	< 0.001
	Follow-ups vs. Baseline^b^	0.001	0.001	
Developmental burden	Baseline	16.90 ± 4.30	16.67 ± 4.11	0.83
	Follow-up 1	11.20 ± 1.49	12.86 ± 4.02	< 0.001
	Follow-up 2	8.80 ± 3.19	12.77 ± 2.21	< 0.001
	Follow-ups vs. Baseline^b^	0.001	0.001	
Physical burden	Baseline	13.40 ± 2.89	13.23 ± 2.71	0.82
	Follow-up 1	9.20 ± 1.63	10.10 ± 3.42	0.007
	Follow-up 2	7.46 ± 2.90	10.30 ± 2.26	< 0.001
	Follow-ups vs. Baseline^b^	0.001	0.001	
Social burden	Baseline	16.13 ± 3.11	16.23 ± 2.99	0.90
	Follow-up 1	11.07 ± 1.31	13.60 ± 3.32	< 0.001
	Follow-up 2	8.70 ± 2.47	13.26 ± 2.06	< 0.001
	Follow-ups vs. Baseline^b^	0.001	0.001	
Emotional burden	Baseline	13.80 ± 4.49	13.96 ± 4.27	0.88
	Follow-up 1	10.30 ± 2.36	12.33 ± 3.82	< 0.001
	Follow-up 2	8.70 ± 2.63	7.80 ± 263	< 0.001
	Follow-ups vs. Baseline^b^	0.13	0.001	

Data are represented as mean ± standard deviations; data are represented as mean ± standard deviations

*CB* Caregiver burden*, FCGs* Family caregivers, *MS* Multiple Sclerosis, *CBI* Caregiver Burden Inventory

^a^*p*-values for comparing scores between the intervention and control groups, at baseline (derived from independent t-test) and at follow-ups (derived from repeated measures ANOVA)

^b^*p*-value for comparing differences between follow-ups and baseline (derived from repeated measures ANOVA)

### Changes in adherence to HPBs in intervention and control groups

Table 4 and Fig. 2 present the results of the within- and between-group changes in the HPLP-II scores (viz. adherence to HPBs) in both groups. Independent-samples t-test results showed that the mean values of total HPLP-II and other subscales had no significant between- group difference at the baseline stage (*p* > 0.05). Repeated measures ANOVA outcomes further revealed that the between-group comparison of the HPLP-II mean scores (viz. total and all dimensions) at both follow-up stages in the intervention group were significantly higher (*p* < 0.001). The within-group comparison via repeated measures ANOVA additionally showed significant differences between the mean values of the HPLP-II and its six dimensions from the baseline to the follow-up 1 & 2 stages in the intervention group (*p* < 0.001). However, no significant within-group changes were observed for the total score of the HPLP-II and its dimensions (viz. health responsibility, physical activity, nutrition, spiritual growth, interpersonal relationships, and stress management) in the control group (*p* = 0.68, *p* = 0.78, *p* = 0.71, *p* = 0.36, *p* = 0.11, *p* = 0.82, *p* = 0.45, respectively). As well, the within-group changes in the HPLP-II mean score in the intervention group increased significantly from 123.53 ± 14.01 to 148.06 ± 15.04.

**Table 4:** Comparison of changes in mean adherence to HPBs of the intervention and control groups (FCGs of patients with MS)

Group		Intervention (*n* = 30)	Control (*n* = 30)	Intervention vs. Control^a^
HPLP-2 (total and individual dimensions)				
Total HPLP-2 score	Baseline	123.53 ± 14.01	118.57 ± 17.23	0.23
	Follow-up 1	131.20 ± 15.97	115.70 ± 18.16	< 0.001
	Follow-up 2	148.06 ± 15.04	119.3 ± 17.64	< 0.001
	Follow-ups vs. Baseline^b^	0.001	0.68	
Health responsibility	Baseline	22.40 ± 2.37	20.96 ± 3.12	0.43
	Follow-up 1	22.26 ± 3.90	21.03 ± 3.43	< 0.001
	Follow-up 2	25.50 ± 3.84	21.50 ± 3.62	< 0.001
	Follow-ups vs. Baseline^b^	0.001	0.78	
Physical activity	Baseline	19.10 ± 3.12	18.43 ± 3.56	0.44
	Follow-up 1	20.13 ± 3.32	17.83 ± 3.40	< 0.001
	Follow-up 2	23.20 ± 2.60	18.33 ± 3.12	< 0.001
	Follow-ups vs. Baseline^b^	0.001	0.71	
Nutrition	Baseline	20.50 ± 2.90	20.13 ± 3.53	0.66
	Follow-up 1	23.76 ± 3.7	20.36 ± 3.77	< 0.001
	Follow-up 2	25.26 ± 2.90	21.36 ± 3.60	< 0.001
	Follow-ups vs. Baseline^b^	0.001	0.36	
Spiritual growth	Baseline	22.40 ± 2.72	21.53 ± 3.09	0.25
	Follow-up 1	23.23 ± 3.86	19.90 ± 4.51	< 0.001
	Follow-up 2	25.16 ± 3.47	21.10 ± 3.55	< 0.001
	Follow-ups vs. Baseline^b^	0.002	0.11	
Interpersonal relationships	Baseline	20.80 ± 2.75	20.17 ± 3.10	0.41
	Follow-up 1	22.90 ± 3.44	19.93 ± 3.14	< 0.001
	Follow-up 2	25.63 ± 2.94	20.40 ± 3.13	< 0.001
	Follow-ups vs. Baseline^b^	0.001	0.82	
Stress management	Baseline	18.33 ± 3.59	17.33 ± 3.71	0.29
	Follow-up 1	18.90 ± 3.76	16.63 ± 2.87	< 0.001
	Follow-up 2	23.30 ± 2.86	14.43 ± 2.95	< 0.001
	Follow-ups vs. Baseline^b^	0.001	0.45	

Data are represented as mean ± standard deviations; data are represented as mean ± standard deviations

*HPBs* Health promoting behavior*, FCGs* Family caregivers, *MS* Multiple Sclerosis, *HPLP-2* Health-promoting lifestyle profile 2

^a^*p*-values for comparing scores between the intervention and control groups, at baseline (derived from independent t-test) and at follow-ups (derived from repeated measures ANOVA)

^b^*p*-value for comparing differences between follow-ups and baseline (derived from repeated measures ANOVA)

### Absolute changes in CB and adherence to HPBs

The linear mixed model was performed to evaluate the absolute changes in CB and adherence to HPBs from the baseline to the follow-up stages by controlling the demographic variables, such as age, gender, marital status, education, and occupation (Table 5). The results demonstrated that the follow-up score for CBI in the intervention group was 8.92 units significantly lower than that in the controls (*p* < 0.001, 95% CI = -12.87 to − 4.97). Besides, the mean score of follow-ups 1 & 2 was 18.92 and 24.97 units significantly lower than that at the baseline stage, respectively (*p* < 0.001). The follow-up score of the HPLP-II in the intervention group was 16.47 units significantly greater than that in the control group (*p* < 0.001, 95% CI = 11.05 to 21.88). The HPLP-II mean score at the follow-up II stage was also 12.55 units significantly higher than that at the baseline stage (*p* < 0.001, 95% CI = 5.95 to 19.15).

**Table 5:** Mixed model with intervention in FCGs of patients with MS

Dependent	Independent		B	SE	95%CI ^a^	*p*-value
CB (CBI)	Group	Intervention	−8.92	2.01	(−12.87, −4.97)	< 0.001**
		Control (ref)				
	Time	Baseline (ref)				
		Follow-up 1	−18.92	2.01	(−22.87, −14.97)	< 0.001**
		Follow-up 2	−24.97	2.84	(−30.54, −19.40)	< 0.001**
adherence to HPBs (HPLP-2)	Group	Intervention	16.47	2.76	(11.05, 21.88)	< 0.001**
		Control (ref)				
	Time	baseline (ref)				
		Follow-up 1	2.40	3.12	(−3.71, 8.51)	0.442
		Follow-up 2	12.55	3.37	(5.95, 19.15)	< 0.001**

Dependent Variable: change of CB and HPLP-2 (adherence to HPBs) from baseline to final intervention

*CB* Caregiver burden, *CBI* Caregiver Burden Inventory, *HPBs* Health promoting behaviors, *HPLP-II* Health-promoting lifestyle profile II

^a^95% confidence intervals; ref.: references category

*p < 0.05, **p < 0.001

## Discussion

This study aimed to investigate the effect of the HLEP on CB and adherence to HPBs in the FCGs of patients with MS, wherein the results demonstrated that involving FCGs in the HLEP could have positive effects on their CB and adherence to HPBs. The study findings also suggested that the FCGs needed adequate education and support regarding health responsibility, physical activity, nutrition, moral development, interpersonal relationships, and stress management to reduce CB and improve adherence to HPBs.

Besides, the results confirmed the first research hypothesis, i.e., the HLEP could have a positive effect on CB in the FCGs of patients living with MS from the baseline to the follow-up stages, in line with the findings of other studies, recruiting various populations. In this regard, one study in Iran had revealed that group-based psychological training programs could lead to a decrease in the CB of the FCGs of patients with MS [24]. Khazaeili et al. had similarly demonstrated that mindfulness-based intervention via a web conferencing app could reduce the burden of the FCGs of MS patients [25]. As well, six months after an intervention entitled the “Resources for Enhancing All Caregivers’ Health Program”, statistically and clinically significant improvements had been reported in depressive symptoms among the FCGs of MS patients in another study, wherein the participants had been bothered by challenging MS behaviors [33]. Lök and Bademli had also found that the program entitled “First You Should Get Stronger”, consisting of some dimensions of HPBs, could significantly relieve CB and develop healthy lifestyle behaviors in the FCGs of dementia patients [28]. Besides, Dehghani et al. had stated that the communication skills training intervention had a significant impact on CB and quality of life in the FCGs of elderly patients with dementia [34]. A randomized controlled trial had correspondingly demonstrated that an individualized physical activity intervention could improve perceived burden among the FCGs of cases with dementia [26]. Since the findings of this study highlighted the positive effect of the HLEP on CB, it is crucial to start developing and implementing such programs for the FCGs of patients living with MS.

The results also confirmed the second research hypothesis about the effectiveness of the HLEP on the FCGs’ adherence to HPBs (viz. health responsibility, physical activity, nutrition, spiritual growth, interpersonal relationships, and stress management), in agreement with similar studies. In this regard, Lök and Bademli had further shown that a health-promoting program had significantly developed adherence to HPBs in the FCGs of dementia patients [28]. Farran et al. had similarly reported that an individualized physical activity intervention could increase physical activity in the FCGs of patients affected with dementia [26]. Besides, another study had suggested that wellness education intervention in patients with stage IV non–small cell lung cancer undergoing icotinib hydrochloride treatment and their FCGs could improve their anxiety and depression as well as quality of life [35]. Since these findings suggested that the HLEP could boost adherence to HPBs, it is essential to design and implement such programs for the FCGs of patients living with MS.

Such findings can be thus valuable because the present study was the first attempt to examine the effect of the HLEP and all its subscales, including health responsibility, nutrition, physical activity, spiritual growth, interpersonal relationships, and stress management on Iranian FCGs of patients with MS in terms of CB and adherence to HPBs. Nevertheless, the most important limitation facing this study was the coincidental occurrence of the COVID-19 pandemic and the virtual implementation of the HLEP via WhatsApp.

## Conclusion

Due to the long-term care for their patients and its much pressure, the FCGs of the patients living with MS are vulnerable and need more attention. The study results suggested that empowerment programs such as the HLEP, covering the dimensions of health responsibility, physical activity, nutrition, spiritual growth, interpersonal relationships, and spiritual growth could be effective, thereby reducing CB and improving adherence to HPBs among the FCGs of MS patients. Health care managers, planners, and providers are thus recommended to start developing and implementing HLEPs for the FCGs of patients living with MS to pave the grounds for decreasing CB and augmenting adherence to HPBs. According to the results of this study, HLEPs can be utilized in planning for the FCGs of patients with other chronic diseases. Furthermore, it is recommended to investigate the impact of similar intervention and distance education programs about healthy lifestyles on health conditions, CB, and adherence to HPBs in different FCGs over longer periods, such as 6 months or 1 year, in future studies.

Since online education may not have high effectiveness compared with face-to-face training, it is suggested to apply the HLEP via face-to-face methods for patients with MS after the pandemic is over.

## Data Availability

Restrictions apply to the availability of these data, which were used under data sharing agreement in the present study, and so are not publicly available. The data are, however, available upon reasonable request from the corresponding author.

## Abbreviations

MS: Multiple Sclerosis
CB: Care burden
FCGs: Family caregivers
HPBs: Health-promoting behaviors
HLEP: Healthy lifestyle empowerment program
HPLP-II: Health-promoting lifestyle profile-II
CBI: Caregiver Burden Inventory
